# Human iPSC-derived motoneurons harbouring *TARDBP* or *C9ORF72* ALS mutations are dysfunctional despite maintaining viability

**DOI:** 10.1038/ncomms6999

**Published:** 2015-01-12

**Authors:** Anna-Claire Devlin, Karen Burr, Shyamanga Borooah, Joshua D. Foster, Elaine M. Cleary, Imbisaat Geti, Ludovic Vallier, Christopher E. Shaw, Siddharthan Chandran, Gareth B. Miles

**Affiliations:** 1 School of Psychology and Neuroscience, University of St. Andrews, Westburn Lane, St. Andrews KY16 9JP, UK; 2 Euan MacDonald Centre for Motor Neurone Disease Research, Edinburgh EH16 4SB, UK; 3 Centre for Neuroregeneration and Medical Research Council Centre for Regenerative Medicine, University of Edinburgh, Edinburgh EH16 4UU, UK; 4 Wellcome Trust-Medical Research Council Stem Cell Institute, Anne McLaren Laboratory for Regenerative Medicine, Department of Surgery, University of Cambridge, Cambridge CB2 0XY, UK; 5 MRC Centre for Neurodegeneration Research, King’s College London, Institute of Psychiatry, De Crespigny Park, London SE5 8AF, UK

## Abstract

Amyotrophic lateral sclerosis (ALS) is a devastating neurodegenerative disease for which a greater understanding of early disease mechanisms is needed to reveal novel therapeutic targets. We report the use of human induced pluripotent stem cell (iPSC)-derived motoneurons (MNs) to study the pathophysiology of ALS. We demonstrate that MNs derived from iPSCs obtained from healthy individuals or patients harbouring *TARDBP* or *C9ORF72* ALS-causing mutations are able to develop appropriate physiological properties. However, patient iPSC-derived MNs, independent of genotype, display an initial hyperexcitability followed by progressive loss of action potential output and synaptic activity. This loss of functional output reflects a progressive decrease in voltage-activated Na^+^ and K^+^ currents, which occurs in the absence of overt changes in cell viability. These data implicate early dysfunction or loss of ion channels as a convergent point that may contribute to the initiation of downstream degenerative pathways that ultimately lead to MN loss in ALS.

## Abstract (web summary)

Amyotrophic lateral sclerosis (ALS) is a neurodegenerative disease that affects spinal cord motor neurons. Here the authors use induced pluripotent stem cell-derived motor neurons obtained from patients with ALS-linked mutations, and find functional deficits resulting from a progressive decrease in voltage-activated Na^+^ and K^+^ currents that occur in the absence of cell death.

## Graphical abstract

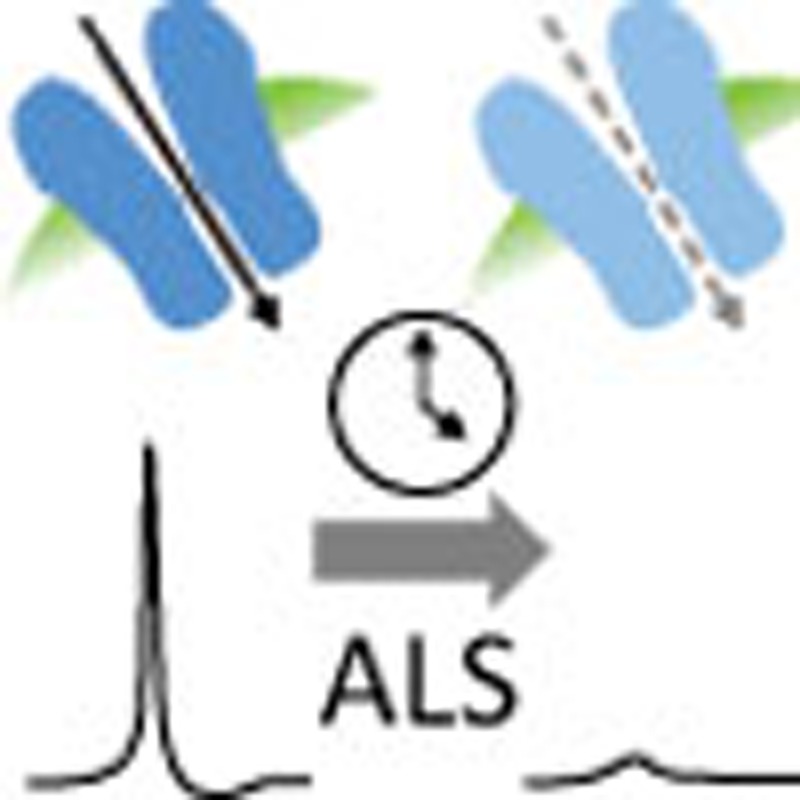

Amyotrophic lateral sclerosis (ALS) is a rapidly progressing, fatal neurodegenerative disease for which no effective treatment exists. Although the mechanisms underlying motoneuron (MN) loss in ALS remain unclear, recent pathological and genetic discoveries have provided important insights that implicate common mechanisms across both ALS and fronto-temporal dementia, as well as a link between familial and sporadic ALS^1^,^2^. The discovery of a common pathological signature characterized by cytoplasmic accumulation of TDP-43 in sporadic forms of fronto-temporal dementia and ALS, along with the discovery of mutations in *TARDBP* and *C9ORF72* in familial and sporadic forms of these diseases, has become increasingly influential in shaping our understanding of ALS and related disorders^3^,^4^,^5^,^6^. Nonetheless, despite the many advances driven by accumulating genetic discoveries there has been a striking failure to translate experimental observations into therapies^7^. This reflects, in part, the absence of appropriate human cell-based models in which to validate disease mechanisms and test candidate therapeutics before lengthy and costly clinical trials. Human induced pluripotent stem cell (iPSC) systems have the potential to bridge this gap. Specifically, they allow us to study the consequences of mutation(s) expressed at disease-relevant levels in functional disease-relevant cell types. We and others have previously reported that iPSC-derived human neurons and astroglia recapitulate key aspects of the adult human pathology and biochemistry that are hallmarks of ALS^8^,^9^,^10^,^11^,^12^,^13^,^14^,^15^. In addition to revealing aspects of the molecular pathology of MNs derived from ALS patients, iPSC-derived neurons may reveal early and subtle pathophysiological changes, which may highlight new targets for therapeutic interventions that ultimately aim to maintain MN function in ALS.

Interestingly, physiological analyses have already revealed changes in the functional properties of MNs at very early stages (embryonic and early postnatal) in transgenic rodent models of ALS. Several studies have demonstrated pre-symptomatic hyperexcitability of MNs due to perturbations in their intrinsic properties, particularly Na^+^ currents^16^,^17^,^18^,^19^,^20^,^21^,^22^. Neuronal hyperexcitability, again thought to relate to changes in Na^+^ currents, has also been shown in studies of ALS patients^23^,^24^,while very recent studies of human iPSC-derived MNs have reported conflicting results of hyperexcitability^14^ versus hypoexcitability^12^. Taken together, these findings suggest that perturbations in the intrinsic biophysical properties of MNs lead to aberrant activity that may reflect and contribute to the earliest events that ultimately lead to irreversible neurodegeneration in ALS. Furthermore, they demonstrate the sensitive nature of electrophysiological studies of MN function and highlight their potential to reveal important early pathogenic mechanisms occurring before molecular or anatomical signs of neurodegeneration.

In the present study, we have undertaken detailed, temporal electrophysiological analyses of human iPSC-derived MNs, to investigate whether MN dysfunction represents an early feature of ALS pathogenesis common to neurons carrying mutations in *TARDBP* and *C9ORF72*. We demonstrate the development of appropriate functional properties in both control and patient iPSC-derived MNs but reveal a progressive loss of action potential output, spontaneous synaptic activity and ionic conductances in patient-derived MNs, regardless of their genotype, which occurs before any overt changes in cell viability.

## Results

### Differentiation of MNs from human iPSC lines

iPSCs were generated from fibroblasts of ALS patients and healthy individuals as previously described^9^,^25^. We used eight iPSC lines: two clones from one *TARDBP* patient (lines D1 and D3); single clones from two different *C9ORF72* patients (lines S6 and R2); two clones from one healthy control (lines R6 and M2); and single clones from two additional healthy controls (lines D6 and D9; see Methods for further details), with each line differentiated a minimum of four times.

**Figure 1:**
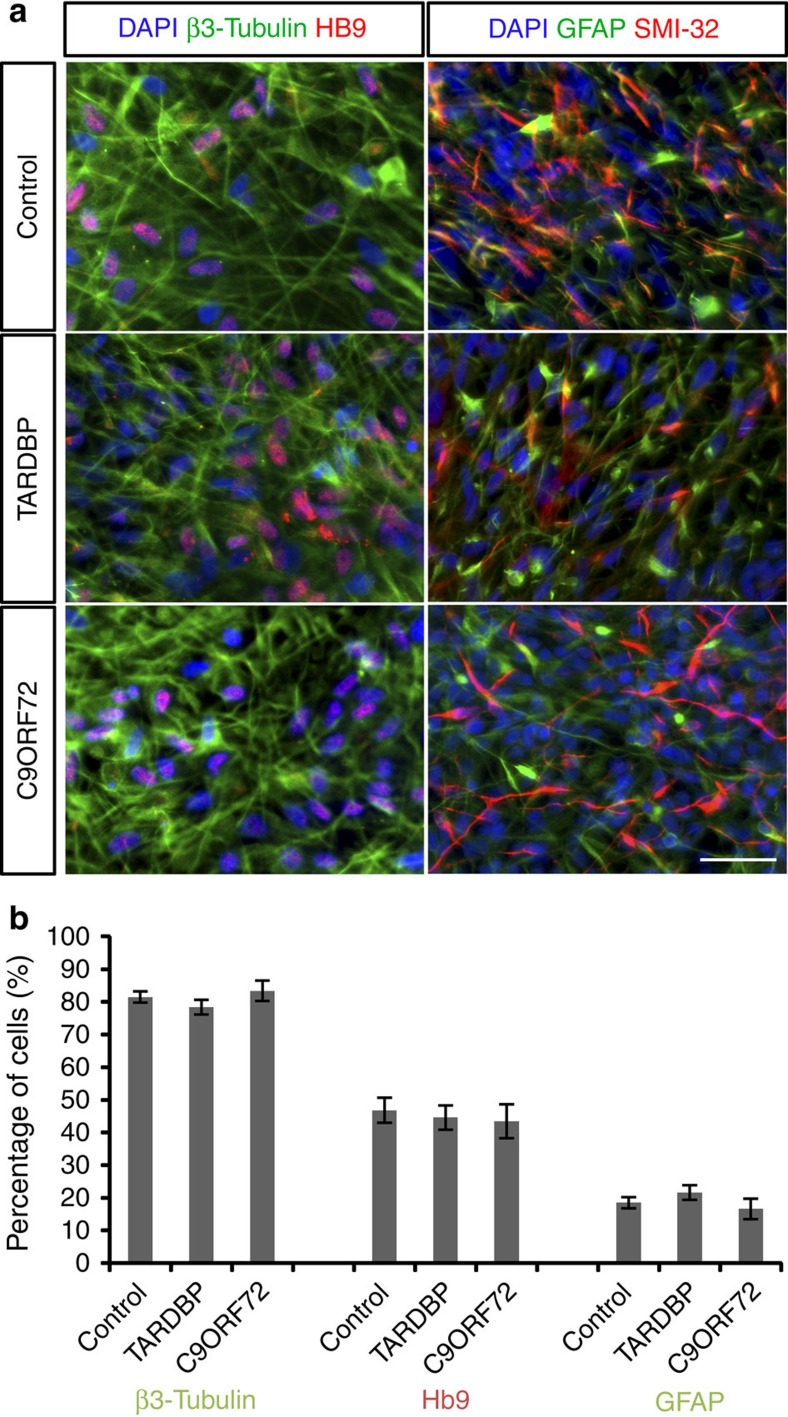
Differentiation of MNs from control and patient iPSC lines. (**a**) Immunohistochemical staining of differentiated iPSCs (control, *TARDBP* and *C9ORF72* lines) using antibodies raised against β3-tubulin, Hb9, SMI-32 and glial fibrillary acidic protein (GFAP). (**b**) Proportion of differentiated iPSCs expressing β3-tubulin, Hb9 and GFAP (total cells counted: control, 1,126 cells; *TARDBP*, 1,224 cells; *C9ORF72*, 1,534 cells; scale bar, 50 μm).

Differentiation into a neuronal and MN lineage was performed using a modified version of established protocols, which enabled the maintenance of cells for up to 10 weeks^9^,^26^. At weeks 5–6 post plating, immunohistochemistry was performed on iPSC-derived MNs, to assess the relative expression of glial, neuronal and MN markers. Quantitative immunolabelling for β3-tubulin and glial fibrillary acidic protein revealed comparable neuronal and astroglial differentiation from one control (D6) and two patient (*TARDBP* D1 and *C9ORF72* S6) iPSC lines (β3-tubulin: Control 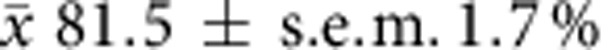, *TARDBP* 78.4±2.2%, *C9ORF72* 83.4±3.1%; glial fibrillary acidic protein: Control 18.5±1.7%, *TARDBP* 21.6±2.2%, *C9ORF72* 16.6±3.1%; Fig. 1a,b), as well as a similar proportion of Hb9-positive MNs (Control 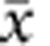 46.8±s.e.m. 3.8%, *TARDBP* 44.6±3.7%, *C9ORF72* 43.4±5.2%; Fig. 1a,b; negative binomial generalized linear model with multiple Wald’s tests and Bonferroni correction). These findings are consistent with previous reports of MN-enriched cultures derived from *TARDBP* iPSC lines^9^.

### Comparable viability of neurons derived from all iPSC lines

**Figure 2:**
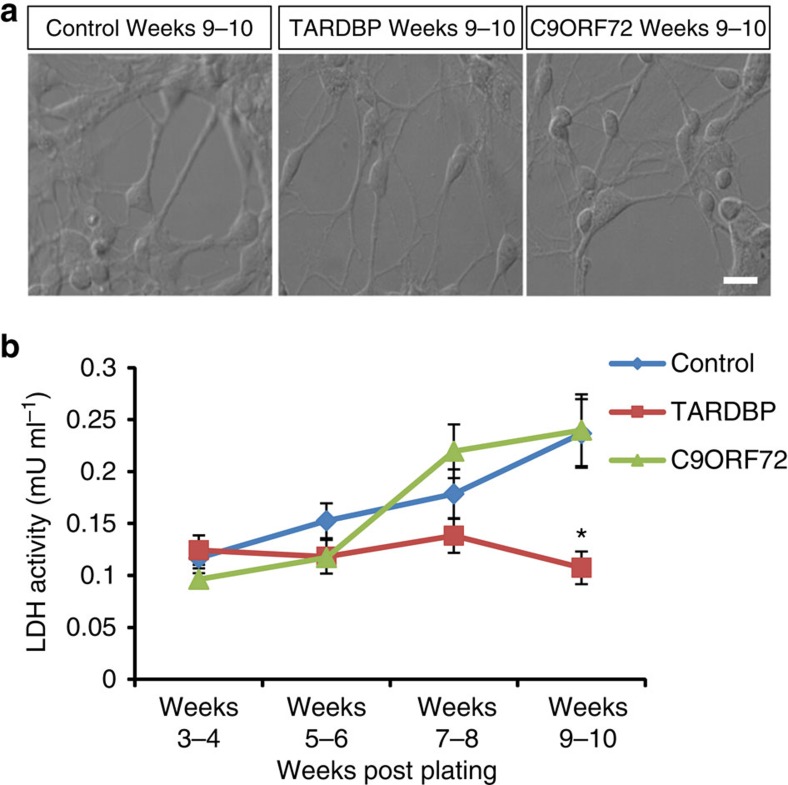
Equivalent viability of control and patient iPSC-derived MNs. (**a**) IR-DIC images of iPSC-derived MNs from control, *TARDBP* and *C9ORF72* lines at weeks 9–10 post plating (scale bar, 20 μm). (**b**) LDH activity plotted for control and patient iPSC-derived cultures from weeks 3–10 post plating (Control lines (D6: two experiments, M2 and R6), *TARDBP* lines (D1 and D3), *C9ORF72* (S6: two experiments and R2); data are plotted as 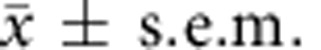; *P<0.05; factorial ANOVA).

Having established equivalent MN-enriched cultures from both control and patient iPSC lines, we first investigated whether there were any differences in cell viability between neurons derived from control and ALS patient iPSCs. Our initial observations suggested that control and patient iPSC-derived neurons with either *TARDBP* or *C9ORF72* ALS mutations were indistinguishable, based on cell morphology (Fig. 2a), up to the latest time point investigated (10 weeks post plating). To confirm this, we performed quantitative analyses of cell viability using cell counts, lactate dehydrogenase (LDH) assays and assessment of nuclear morphology. Cell counts performed on infrared-differential interference contrast (IR-DIC) images revealed no difference between patient-derived lines compared with controls throughout the 10 weeks in culture (80 images analysed per cell type; Fig. 2a). Analysis of LDH activity in two *TARDBP* lines (D1 and D3), two *C9ORF72* lines (S6 and R2) and three control lines (D6, M2 and R6) revealed no greater LDH activity in patient compared with control lines at any time point during the 10 weeks in culture (Fig. 2b; factorial analysis of variance (ANOVA)). There was in fact less LDH activity in *TARDBP* lines at 9–10 weeks post plating (Fig 2b, *P*<0.05). Finally, we also assessed nuclear morphology at 9–10 weeks post plating in one control (D6), one *TARDBP* (D1) and one *C9ORF72* (S6) line. We found no difference in the percentage of cells with pyknotic nuclei in patient lines compared with controls (Control 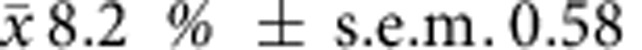, *n*=3,386 cells; *TARDBP* 8.4%±0.25, *n*=1,558; *C9ORF72* 5.4%±0.36, *n*=2,336; Supplementary Fig. 1; one-way ANOVA and Tukey’s *post-hoc* test). In summary, cell counts, LDH assays and counts of pyknotic nuclei failed to reveal any differences in viability between neurons derived from iPSC lines harbouring *TARDBP* or *C9ORF72* mutations compared with controls.

### Functional perturbations in patient iPSC-derived MNs

Given that standard cell viability assays failed to reveal overt effects of *TARDBP* or *C9ORF72* mutations on iPSC-derived cell viability, we next turned to more sensitive electrophysiological analyses to uncover any signs of neuronal dysfunction.

**Figure 3:**
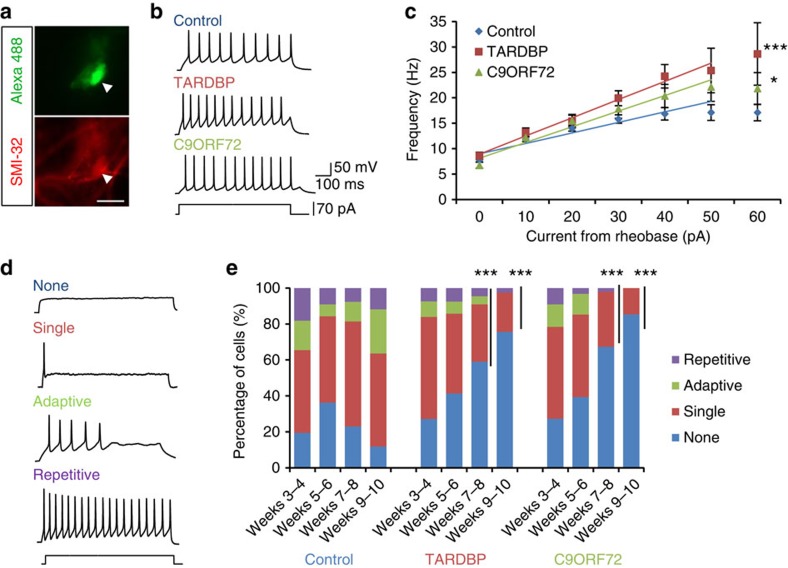
Hyperexcitability followed by loss of action potential output in patient iPSC-derived MNs. (**a**) Fluorescent image of a cell filled with Alexa Fluor 488 dye during whole-cell patch-clamp recordings and immunolabelling with an antibody raised against SMI-32 (arrow heads point to cell soma; scale bar, 10 μm). (**b**) Repetitive firing in response to square current injection in iPSC-derived MNs from control, *TARDBP* and *C9ORF72* lines. (**c**) Frequency-current (*f–I*) relationships generated for repetitively firing iPSC-derived MNs from control (*n*=62), *TARDBP* (*n*=19) and *C9ORF72* (*n*=19) lines recorded from weeks 2–6 post plating (data are plotted as 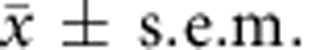 with lines of best fit; *significantly different to control, *P*<0.05; ***significantly different to control, *P*<0.0001; linear model with multiple contrast for the gradient values, and adjusted with Bonferroni correction). (**d**) Examples of the four categories of firing observed in iPSC-derived MNs (repetitive, adaptive, single or no firing). (**e**) Proportion of cells exhibiting each firing category in iPSC-derived MNs from control (*n*=702), *TARDBP* (*n*=380) and *C9ORF72* (*n*=239) lines across weeks 3–10 post plating (***significantly different to control, *P*<0.0001; logistic regression with multiple Wald’s test and Bonferroni correction).

Whole-cell patch-clamp recordings were obtained from the largest neurons visualized via IR-DIC microscopy in cultures from 2 to 10 weeks post plating. Our selection of the largest neurons for recordings along with the high degree of MN enrichment ensured studied cells were predominantly MNs. In a subset of experiments, this was supported by *post-hoc* SMI-32 labelling of neurons filled with an Alexa Fluor dye during recordings (Fig. 3a). We found that a high proportion of recovered neurons (78%; 18 of 23 cells) were SMI-32 positive. We cannot exclude the possibility that some recordings were from spinal interneurons. However, data from mixed neuronal populations are also valuable given that ALS affects a wide range of neuronal cell types including cortical neurons and spinal interneurons^27^.

We began by comparing the passive membrane properties of MNs derived from control and patient iPSCs. For these and all other electrophysiological analyses, data were pooled for control (D9, D6, R6 and M2), *TARDBP* (D1 and D3) and *C9ORF72* (R2 and S6) iPSC lines (see Supplementary Table 1 and Supplementary Fig. 2 for data and sample sizes separated by iPSC line). Whole-cell capacitance (*C*_m_) values were similar across control and patient iPSC-derived MNs (see Table 1 for 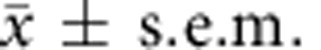 and sample sizes; factorial ANOVA with Tukey’s honest significant difference), although MNs harbouring *C9ORF72* mutations had lower *C*_m_ compared with MNs harbouring *TARDBP* mutations at weeks 3–4, weeks 7–8 and weeks 9–10 (*P*<0.001), and control MNs had lower *C*_m_ compared with MNs harbouring *TARDBP* mutations at weeks 7–8 and weeks 9–10 (*P*<0.0001). We found no significant differences in the input resistance (*R*_N_) of MNs derived from control, *TARDBP* or *C9ORF72* iPSCs from weeks 3 to 10 post plating (Table 1). MNs harbouring a *TARDBP* mutation had more depolarized resting membrane potentials (RMPs) compared with controls at weeks 3–4 (*P*<0.001) and weeks 9–10 (*P*<0.0001), while the RMPs of MNs with a *C9ORF72* mutation were more depolarized than controls at weeks 7–8 (Table 1; *P*<0.0001). The largest differences in RMP between control and patient iPSC-derived MNs were apparent towards the end of the time course studied. It should be noted that these numbers may represent an underestimation of the extent of membrane potential depolarization in patient iPSC-derived MNs, because cells that were more depolarized than −20 mV were excluded from all analyses, to avoid the inclusion of cells that were depolarized due to damage associated with the establishment of recordings.

Current-clamp mode recordings of iPSC-derived MNs demonstrated that by 2 weeks post plating, all lines developed the ability to repetitively fire trains of action potentials in response to current injections. To compare the excitability of repetitively firing patient and control iPSC-derived MNs, frequency–current (*f*–*I*) relationships were generated from responses to a series of injected current steps (0 to 145 pA, in 10 pA increments, 1 s duration). Comparisons were performed on data pooled from recordings of repetitively firing cells at weeks 2–6 post plating (Fig. 3b,c). No differences were observed in rheobase current between control and patient iPSC-derived MNs (Control 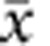 22.7±s.e.m. 2.6 pA, *n*=62; *TARDBP* 23.4±4.1 pA, *n*=19; *C9ORF72* 17.1±3.2 pA, *n*=19; one-way ANOVA). However, data demonstrated hyperexcitability in both the *TARDBP* and *C9ORF72* lines compared with controls, as indicated by the greater slopes of their *f*–*I* relationships (Control 0.20±s.e. 0.01 Hz pA^−1^, *TARDBP* 0.36±0.04 Hz pA^−1^, *C9ORF72* 0.31±0.03 Hz pA^−1^; *P*<0.05; linear model with multiple contrast for gradient values, and adjusted with Bonferroni correction). No significant difference was detected between the excitability of MNs derived from the *TARDBP* and *C9ORF72* lines. Thus, in parallel with findings in animal models of ALS, human MNs harbouring two different ALS-related mutations are initially more excitable than controls.

Not all iPSC-derived MNs produced repetitive trains of action potentials, demonstrating an incomplete functional maturation in some neurons. Several different output patterns were observed in response to current injections, which included repetitive, adaptive, single and no firing (Fig. 3d). Repetitive firing was defined as a train of action potentials that lasted for the duration of the square current injection (1 s), while adaptive firing was defined as multiple action potentials that ceased before the end of the current stimuli. Cells were classified as adaptive if they were unable to repetitively fire in response to any of the series of current steps applied. When data were compared across MNs derived from control, *TARDBP* and *C9ORF72* iPSC lines, no difference in the relative proportion of firing versus non-firing cells were found from weeks 3 to 6 post plating (Control firing 70.4%, *n*=419, *TARDBP* firing 64.0%, *n*=214; *C9ORF72* firing 67.7%, *n*=149; Fig. 3e; logistic regression with multiple Wald’s tests and Bonferroni correction). However, at weeks 7–8 and 9–10 post plating, the number of cells able to fire action potentials decreased significantly in *TARDBP* and *C9ORF72* lines compared with controls (*P*<0.0001), while the ratio of firing versus non-firing cells remaining unchanged throughout the 10 weeks in control lines (Weeks 7–8: control firing 77.0%, *n*=183; *TARDBP* firing 40.9%, *n*=88; *C9ORF72* firing 32.6%, *n*=49; Weeks 9–10: control firing 88.1%, *n*=110; *TARDBP* firing 24.3%, *n*=78; *C9ORF72* firing 14.6%, *n*=41; Fig. 3d). These data demonstrate a loss of action potential output from iPSC-derived MNs harbouring ALS-related mutations, which renders them non-functional despite their continued viability in culture.

### Loss of synaptic activity in patient iPSC-derived MNs

**Figure 4:**
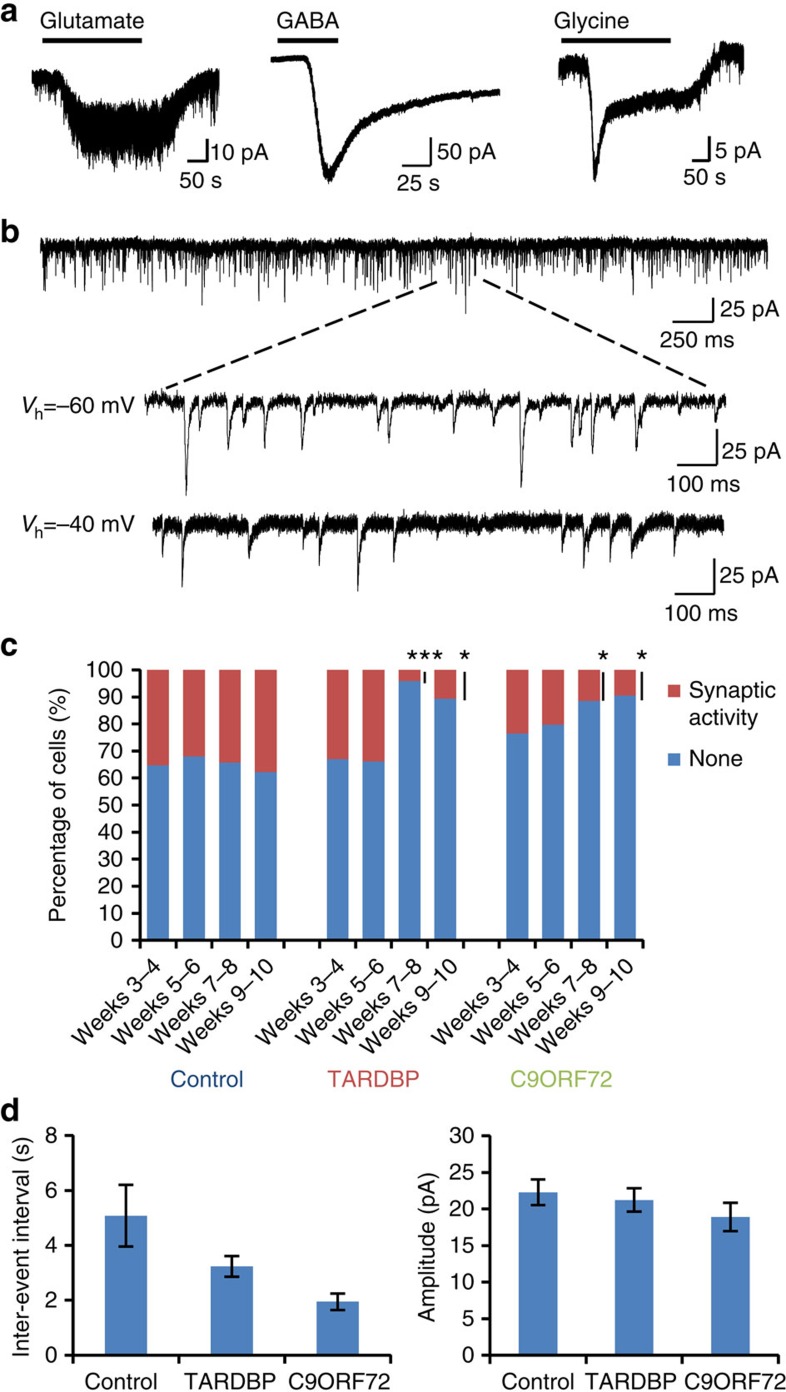
Loss of synaptic input to patient iPSC-derived MNs. (**a**) Current responses in iPSC-derived MNs during bath application of glutamate (100 μM), GABA (100 μM) and glycine (100 μM). (**b**) Voltage-clamp recordings of spontaneous synaptic activity in iPSC-derived MNs at different holding potentials. (**c**) Proportion of cells displaying synaptic activity from weeks 3–10 post plating in iPSC-derived MNs from control (*n*=845), *TARDBP* (*n*=417) and *C9ORF72* (*n*=265) lines. (*significantly different to control, *P*<0.05; ***significantly different to control, *P*<0.0001; logistic regression with multiple Wald’s tests and Bonferroni correction). (**d**) Graphs of inter-event interval and amplitude of synaptic events recorded from control and patient iPSC-derived MNs.

Following investigation of the output produced by iPSC-derived MNs, we next examined their ability to receive synaptic input, which is reported to be attenuated in ALS patients^28^,^29^,^30^ and animal models of the disease^31^,^32^,^33^. We first assessed whether iPSC-derived MNs could respond to the major excitatory neurotransmitter (NT) glutamate (100 μM) or the inhibitory NTs GABA (100 μM) and glycine (100 μM). This was assessed by bath applying these NTs during voltage-clamp recordings from iPSC-derived MNs held at −60 mV (Fig. 4a). Depolarizing currents were recorded from MNs derived from both control and patient iPSC lines in response to glutamate applications (Fig. 4a). Control and patient iPSC-derived MNs also responded to GABA and glycine (Fig. 4a) with depolarizing currents as expected, based on the theoretical reversal potential for chloride in our recording solutions. Thus, both control and patient iPSC-derived MNs developed receptors required to respond to major NTs.

Next, we assessed whether iPSC-derived MNs received functional synaptic inputs in culture. Spontaneous synaptic activity was observed in our recordings of both control and patient iPSC-derived MNs throughout the time period studied (Fig. 4b). Consistent with previous reports^9^,^14^, these inputs were predominantly excitatory as evidenced by a lack of outward currents, even when cells were held at −40 mV, which is well above the reversal potential for chloride (Fig. 4b). The proportion of cells that received any synaptic input (defined as at least 1 event per minute) was similar in control and patient iPSC-derived MNs from weeks 3–6 post plating (3–6 weeks: 33.2% of controls, *n*=518; 33.5% of *TARDBP*, *n*=274; 22.2% of *C9ORF72*, *n*=171; Fig. 4c; logistic regression with multiple Wald’s tests and Bonferroni correction). The occurrence of synaptic activity then decreased in both patient-derived lines from weeks 7–10 compared with controls (7–8 weeks: 34.2% of controls, *n*=216; 4.1% of *TARDBP*, *n*=96; 11.5% of *C9ORF72*, *n*=52; 9–10 weeks: 37.8% of controls, *n*=111; 6.1% of *TARDBP*, *n*=82; 9.5% of *C9ORF72*, *n*=42; *P*<0.05; Fig. 4c). In cells cultured from 2–6 weeks, we analysed the inter-event-interval and amplitude of synaptic events. Neither parameter differed between controls and patient iPSC-derived MNs (Fig. 4d). In summary, these data demonstrate a loss of synaptic input to *TARDBP* and *C9ORF72* iPSC-derived MNs, which may reflect the parallel loss of action potential output in these cultures or perturbations in synaptic transmission between cultured cells.

### Loss of voltage-gated currents in patient iPSC-derived MNs

To investigate mechanisms underlying the progressive loss of action potential output from patient iPSC-derived MNs, we next performed voltage-clamp recordings of voltage-activated currents involved in action potential generation.

**Figure 5:**
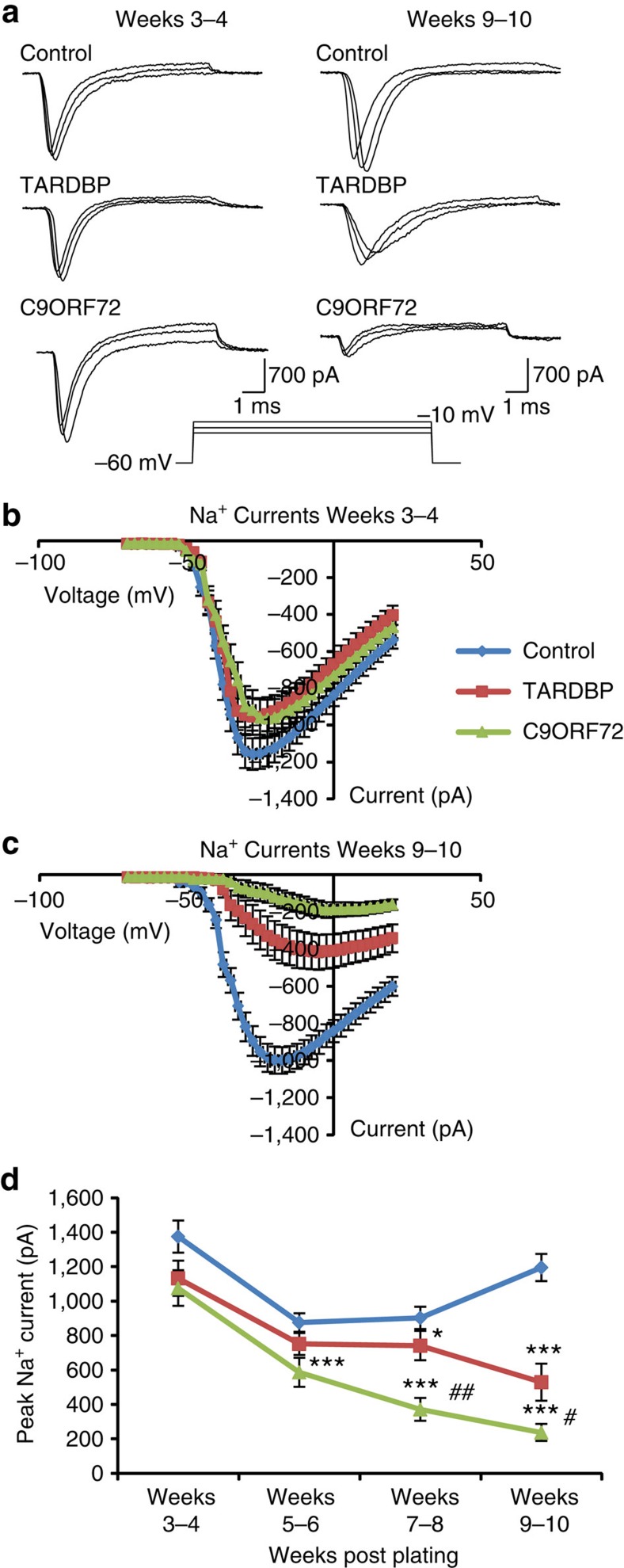
Loss of fast-inactivating Na^+^ currents in patient iPSC-derived MNS. (**a**) Raw data showing fast, inactivating Na^+^ currents in control, *TARDBP* and *C9ORF72* iPSC-derived MNs at week 3–4 and weeks 9–10. (**b**) Current–voltage relationships of peak Na^+^ currents recorded from control and patient iPSC-derived MNs at weeks 3–4. (**c**) Current–voltage relationships of peak Na^+^ currents recorded from control and patient iPSC-derived MNs at weeks 9–10. (**d**) Peak fast, inactivating Na^+^ currents plotted from weeks 3–10 for control (*n*=847), *TARDBP* (*n*=452) and *C9ORF72* (*n*=264) iPSC-derived MNs. (data are plotted as 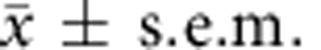; *significantly different to controls, *P*<0.05; ***significantly different to controls, *P*<0.0001; #significant difference between patient lines, *P*<0.05; ##significant difference between patient lines, *P*<0.001; linear model with multiple Wald’s tests and Bonferroni correction).

We first investigated fast, inactivating Na^+^ currents (Fig. 5a), which underlie the upstroke of the action potential, by using a series of voltage steps (−70 to 20 mV, 2.5 mV increments, 10 ms duration) from a holding potential of −60 mV (Fig. 5b,c). We found no differences in the current–voltage (*I*–*V*) relationships or peak Na^+^ currents between MNs derived from patient and control iPSCs at weeks 3–4 post plating (Peak current: 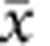 control 1,375±s.e.m. 93 pA, *n*=196; *TARDBP* 1,132±101 pA, *n*=103; *C9ORF72* 1,075±103 pA, *n*=102; Fig. 5b,d; linear model with multiple Wald’s tests and Bonferroni correction). However, from week 5 post plating, we observed a progressive loss of Na^+^ currents in MNs derived from patient iPSCs. Peak Na^+^ currents were reduced compared with controls from weeks 5–10 post plating in *C9ORF72* iPSC-derived MNs (*P*<0.0001) and from weeks 7–10 post plating in *TARDBP* iPSC-derived MNs (*P*<0.01), while peak currents were also smaller in neurons derived from *C9ORF72* iPSCs compared with those derived from *TARDBP* iPSCs (*P*<0.05) from week 7 onwards (weeks 5–6: 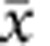 control 875±s.e.m. 53 pA, *n*=322; *TARDBP* 751±64 pA, *n*= 171; *C9ORF72* 587±84 pA, *n*=69; weeks 7–8: control 902±64 pA, *n*=216; *TARDBP* 741±85 pA, *n*=96; *C9ORF72* 371±66 pA, *n*=52; weeks 9–10: control 1,196±78 pA, *n*=111; *TARDBP* 529±108 pA, *n*=82; *C9ORF72* 236±49 pA, *n*=42; Fig. 5a,c,d).

**Figure 6:**
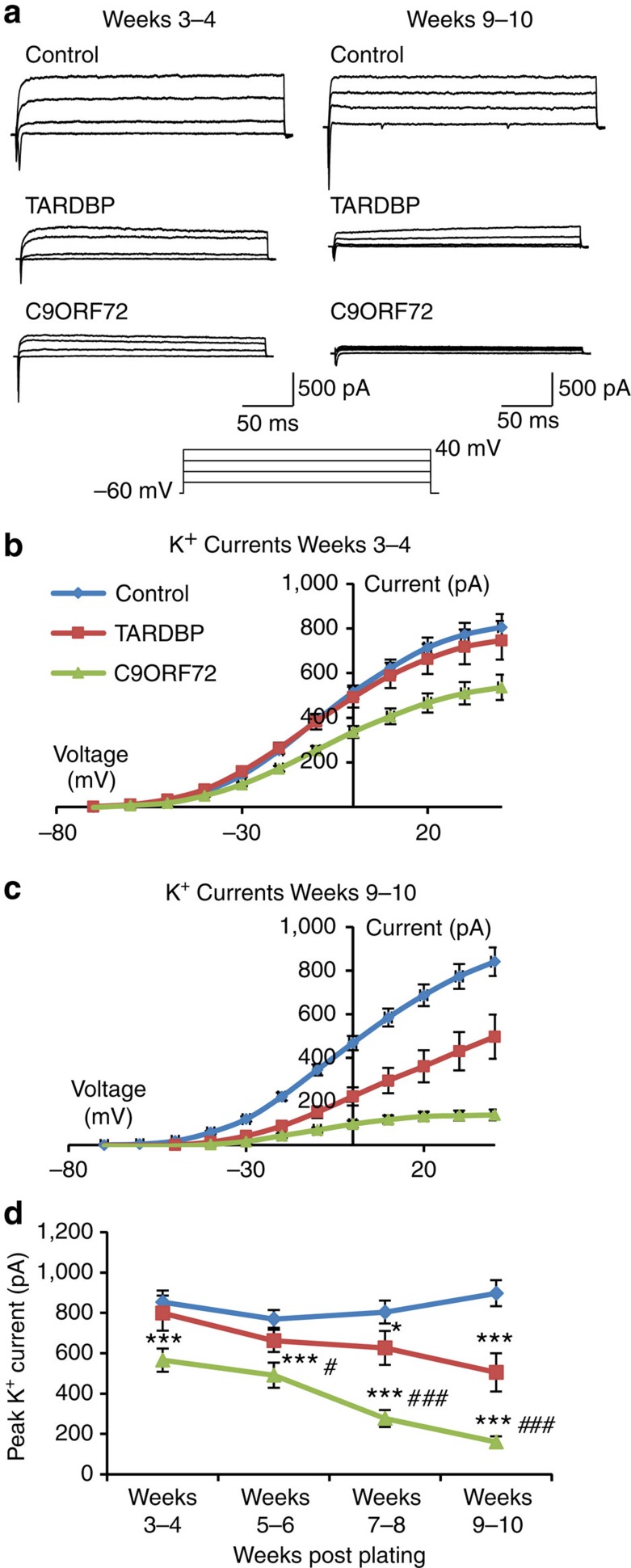
Loss of persistent voltage-activated K^+^ currents in patient iPSC-derived MNs. (**a**) Raw data showing persistent K^+^ currents in control, *TARDBP* and *C9ORF72* iPSC-derived MNs at week 3–4 and weeks 9–10. (**b**) Current–voltage relationships of peak K^+^ currents recorded from control and patient iPSC-derived MNs at weeks 3–4. (**c**) Current–voltage relationships of peak K^+^ currents recorded from control and patient iPSC-derived MNs at weeks 9–10. (**d**) Peak K^+^ currents plotted from weeks 3–10 for control (*n*=847), *TARDBP* (*n*=452) and *C9ORF72* (*n*=264) iPSC-derived MNs (data are plotted as 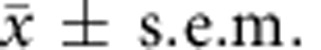; *significantly different to control, *P*<0.05; ***significantly different to control, *P*<0.0001; #significant difference between patient lines, *P*<0.05; ###significant difference between patient lines, *P*<0.0001; linear model with multiple Wald’s tests and Bonferroni correction).

Having revealed a progressive loss of Na^+^ currents, we next assessed whether this reflected a more general decrease in voltage-activated currents in patient iPSC-derived MNs. This was achieved by measuring persistent K^+^ currents (Fig. 6a) elicited by a series of voltage steps (−70 to 40 mV, 10 mV increments, 500 ms duration) from a holding potential of −60 mV (Fig. 6b,c). At weeks 3–4 post plating, peak K^+^ currents were comparable in *TARDBP* and control iPSC-derived MNs but were smaller in MNs derived from *C9ORF72* iPSCs when compared with controls (Peak current: 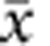 control 853±s.e.m. 57 pA, *n*=196; *TARDBP* 799±86 pA, *n*=103; *C9ORF72* 565±57 pA, *n*=102; Fig. 6a,b,d; linear model with multiple Wald’s tests and Bonferroni correction). We then observed a progressive decline in peak K^+^ currents in patient iPSC-derived MNs, with MNs harbouring a *C9ORF72* mutation having significantly smaller K^+^ currents than controls at all time points (*P*<0.0001) and MNs derived from *TARDBP* iPSCs having smaller K^+^ currents compared with controls from weeks 7–8 post plating onwards (weeks 5–6: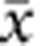 control 769±s.e.m. 44 pA, *n*=322; *TARDBP* 661±54 pA, *n*=171; *C9ORF72* 490±61 pA, *n*=69; weeks 7–8: control 804±57 pA, *n*=216; *TARDBP* 626±84 pA, *n*=96; *C9ORF72* 276±41 pA, *n*=52; weeks 9–10: control 897±64 pA, *n*=111; *TARDBP* 505±95 pA, *n*=82; *C9ORF72* 158 ±29 pA, *n*=42; *P*<0.05; Fig. 6a,c,d). K^+^ currents were also smaller in MNs derived from *C9ORF72* compared with *TARDBP* iPSCs from weeks 5–6 post plating onwards (*P*<0.05).

Taken together, these data demonstrate a progressive reduction in both fast, inactivating Na^+^ currents and persistent, voltage-activated K^+^ currents in patient iPSC-derived MNs. Given the similar time courses of current loss and action potential loss, and the observation that current loss precedes changes in the probability of firing, it is likely to be that reductions in voltage-activated currents underlie the progressive loss of functional output in MNs harbouring ALS-related mutations.

**Figure 7:**
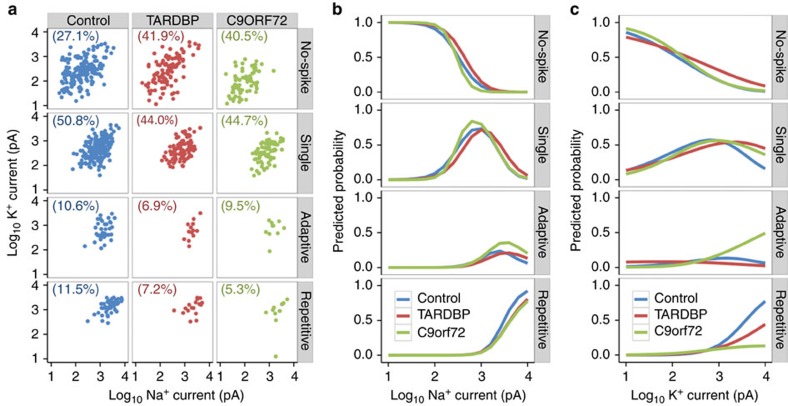
Firing categories of iPSC-derived MNs are predicted by peak Na^+^ and K^+^ currents. (**a**) Relationship between peak Na^+^ and K^+^ currents and action potential firing category were determined using multinomial logistic regressions in control (*n*=448), *TARDBP* (*n*=230) and *C9ORF72* (*n*=176) iPSC-derived MNs at 3–6 weeks post plating. Values in parentheses denote the proportion, as a percentage, of iPSC-derived MNs exhibiting each firing category. (**b**) Predicted probability of each firing category calculated over a range of peak Na^+^ currents. (**c**) Predicted probability of each firing category calculated over a range of peak K^+^ currents.

To examine this further, we investigated the relationship between the magnitude of voltage-activated Na^+^ and K^+^ currents, and the type of output produced by iPSC-derived MNs for which both voltage- and current-clamp data were available (control, *n*=573; *TARDBP*, *n*=277; *C9ORF72*, *n*=190). Plots of raw data indicated similar relationships between peak currents and firing categories in all iPSC lines 3–6 weeks post plating; larger currents were associated with greater output (Fig. 7a). To assess this relationship further, we fitted multinomial logistic regressions to the data using the firing categories (No Spike/Single/Adaptive/Repetitive) as the outcome variables with the type of iPSC line (control, *TARDBP* or *C9ORF72*) and peak current (Na^+^ or K^+^) as the predictor variables. Using these models, we assessed the predicted probabilities for each firing category based on the measured currents. Models showed that both Na^+^ and K^+^ currents were strong predictors of firing category (Na^+^ model, *P*<0.001; K^+^ model, *P*<0.001). In addition, the type of iPSC line contributed to the predicted firing outcome (Na^+^ model, *P*<0.01; K^+^ model, *P*<0.05). As expected, we found that the probability of a cell failing to spike in response to current injection decreases with increasing peak Na^+^ or K^+^ currents (No Spike category; Fig. 7b,c). In comparison, the probability of a cell exhibiting adaptive or repetitive firing in response to current injection increases with increasing peak Na^+^ or K^+^ currents (Adaptive and Repetitive categories; Fig. 7b,c). Whereas the probability of a cell exhibiting only single spikes is highest at an intermediary value of peak Na^+^ or K^+^ currents (Single category; Fig. 7b,c). Overall, the modelled probabilities suggest an ordered response of firing outcome when Na^+^ or K^+^ currents are increased (No Spike<Single<Adaptive<Repetitive; Fig. 7b,c).

In summary, these findings suggest that the mode of iPSC-derived MN firing (Single/Adaptive/Repetitive) is governed by the size of Na^+^ and K^+^ currents. Therefore, the reduced probability of spiking observed in patient iPSC-derived MNs (Fig. 7c) is likely to reflect perturbations in Na^+^ and K^+^ currents as a consequence of the *TARDBP* and *C9ORF72* mutations they harbour.

## Discussion

We have demonstrated that MNs derived from human iPSCs obtained from healthy individuals or patients harbouring *TARDBP* or *C9ORF72* ALS mutations develop appropriate physiological properties. However, temporal analysis revealed that patient iPSC-derived MNs display an initial hyperexcitability, followed by a progressive loss in action potential output and synaptic activity. This loss of functional output appears to result from a progressive decrease in voltage-activated Na^+^ and K^+^ currents that occurs in the absence of overt changes in cell viability. These novel data from ALS-affected human MNs indicate that early dysfunction or loss of ion channels may contribute to the initiation of downstream degenerative pathways that ultimately lead to MN loss in ALS.

Given that all neurons must be by definition excitable, it is critical that protocols established for the derivation of MNs from iPSCs are validated via the electrophysiological demonstration of appropriate functional properties. Previous studies have demonstrated the ability of human iPSC-derived MNs to fire action potentials in response to current injection and to receive spontaneous synaptic inputs^9^,^12^,^13^,^14^,^26^,^34^. In this study, we used both current- and voltage-clamp recordings to not only demonstrate that iPSC-derived MNs can develop appropriate output and receive synaptic input, but to also investigate the temporal profile of voltage-activated currents underlying these functional properties. We observed comparable rates of morphological and physiological maturation in control and patient iPSC-derived MNs, with all lines reaching equivalent maturity ~3 weeks post plating. These analyses provide a detailed validation of the phenotype of iPSC-derived MNs as well as enabling more sensitive functional comparisons between control and patient iPSC-derived MNs. Given that the ultimate goal of ALS therapeutics is to preserve MN function, it is critical that detailed functional analyses of iPSC-derived MNs are performed alongside more common analyses of cellular pathology such as protein aggregation, RNA accumulation and changes in gene expression^9^,^11^,^12^,^13^,^14^,^15^,^34^,^35^.

Previous studies of ALS patients^23^,^24^,^36^, human iPSCs^14^ and animal models of the disease^16^,^17^,^18^,^19^,^20^,^21^,^22^ have reported early hyperexcitability of spinal MNs and corticospinal neurons of the motor cortex. In this study, we have shown that the most functionally mature (repetitive firing) MNs derived from patient iPSCs harbouring *TARDBP* or *C9ORF72* mutations also exhibit hyperexcitability at early stages in culture. Although hyperexcitability was transient in our study and does not persist into symptomatic stages in animal models of ALS^22^,^37^, an initial phase of increased activity might contribute to and/or trigger a cascade of excitotoxic disease mechanisms involving pathological changes in Ca^2+^ handling^38^,^39^, accumulation of intracellular Ca^2+^ and the eventual activation of cell death pathways. In opposition to this, however, recent work supports a link between hyperexcitability and neuroprotection^40^, in particular when hyperexcitability is induced via activation of cholinergic C-bouton inputs to MNs^41^,^42^, which are known to be enlarged presymptomatically in ALS model mice^43^,^44^. These data suggest that MN hyperexcitability might represent an early compensatory mechanism in ALS-affected MNs. To determine the true role of hyperexcitability in ALS, it will be important for future studies to examine the effects of finely controlled manipulations of excitability on human MNs.

Following an early stage of hyperexcitability, we found that patient iPSC-derived MNs progressively lost their ability to generate action potentials. This was evidenced by a reduction in the proportion of patient iPSC-derived MNs, which were able to fire repetitive or even single spikes. In comparison, the proportion of control iPSC-derived MNs in each of the firing categories remained unchanged throughout the 10 weeks studied. Thus, despite remaining viable in culture, ALS-affected MNs were gradually rendered non-functional. Our findings of a progressive loss of MN output are consistent with recent reports of hypoexcitability of iPSC-derived MNs harbouring a *C9ORF72* mutation^12^ and reduced output of spinal MNs in mSOD1 mice^37^. The latter study, which involved *in vivo* recordings from presymptomatic mSOD1 mice, showed that a significant proportion of MNs could not discharge repetitively in response to current ramps, although their neuromuscular junctions remained functional. Thus, dysfunction at the MN cell body appears to precede that at the neuromuscular junction. Taken together, these findings highlight the importance of addressing early perturbations in mechanisms underlying spike generation at the MN soma when considering disease pathogenesis and potential treatment strategies for ALS.

Importantly, we believe our work has addressed an apparent contradiction arising from recent studies performing similar electrophysiological analysis of ALS patient iPSC-derived MNs. Wainger *et al*.^14^ recently reported hyperexcitability of iPSC-derived MNs harbouring a *SOD-1* mutation at ~4 weeks post plating, a time point equivalent to when we also observed hyperexcitability in iPSC-derived MNs harbouring *TARDBP* or *C9ORF72* mutations. In contrast, Sareen *et al*.^12^ reported hypoexcitability in iPSC-derived MNs harbouring *C9ORF72* mutations at a time point comparable to our week 7–8 post plating when we first observed loss of MN output. Thus, these recent studies support our observation of a progression from hyperexcitability to hypoexcitability. Although this progression is consistent with work in animal models^37^, it should be noted that iPSCs represent a developmental model that may be difficult to directly compare to ageing *in vivo*. It also remains unclear whether mechanistic links exist between hyperexcitability and hypoexcitability, which will be important to address in future studies.

Although our focus was on the intrinsic properties of MNs, we also observed a concomitant reduction in the proportion of patient iPSC-derived MNs that received synaptic inputs. Loss of synaptic activity may simply reflect a general loss of action potential generation in culture. However, given evidence of loss and dysfunction of synapses in ALS^28^,^29^,^30^,^31^,^32^,^33^, specific deficits in synaptic transmission might also contribute to reductions in synaptic activity recorded from patient iPSC-derived MNs. Although we demonstrated that iPSC-derived MNs expressed postsynaptic receptors required to receive input, we were unable to compare the magnitude of responses to NTs across control and patient iPSC-derived MNs due to difficulties in obtaining long-term recordings, as required for drug applications, from sufficient numbers of cells. It will be interesting in future studies, focussed on synaptic function, to investigate the possibility that either pre- or postsynaptic machinery is altered in cultures from patient iPSCs.

Following the demonstration of reduced output from patient iPSC-derived MNs, we used voltage-clamp recordings to investigate the mechanisms likely to underlie this loss of function. Previous studies have demonstrated changes in both the density and function of Na^+^ and K^+^ channels in animal models of ALS^16^,^17^,^21^,^45^, ALS patients^36^,^46^,^47^ and patient iPSC-derived MNs^12^,^14^. Our analyses revealed a progressive loss of voltage-activated Na^+^ (fast, inactivating) and K^+^ (persistent) currents in patient iPSC-derived MNs. Given that current loss began before and continued in parallel with changes in firing probability, and that Na^+^ and K^+^ current magnitudes are excellent predictors of firing patterns, reductions in voltage-activated currents most likely underlie the loss of functional output observed in patient iPSC-derived MNs. Mechanisms underlying the initial hyperexcitability of MNs are less clear. Wainger *et al*.^14^ proposed that reductions in delayed rectifier K^+^ currents underlie hyperexcitability in iPSC-derived MNs harbouring *SOD-1* mutations. Although we also observed lower K^+^ current magnitude in MNs harbouring *C9ORF72* mutations at hyperexcitable stages, the same was not true for MNs harbouring *TARDBP* mutations. Thus, reductions in K^+^ currents are unlikely to be the sole mechanism underlying hyperexcitability in patient iPSC-derived MNs. Either loss or dysfunction of channels may underlie the loss of current observed in patient iPSC-derived MNs. Regardless of the exact effect on ion channels, our data are consistent with the recent proposal that ALS involves channelopathy-like mechanisms, a view which stemmed from the demonstration that mutant SOD1 inhibits the conductance of a mitochondrial ion channel^48^. Interestingly, although ALS-related mutations may cause a general loss of voltage-activated currents, the lack of change in input resistance in patient iPSC-derived MNs indicates that leak channels are unaffected. Furthermore, depolarization of the RMP, again with no changes in input resistance, suggests additional membrane proteins are affected, one possibility being the Na^+^/K^+^ pump for which reduced expression has been shown in mSOD1 mice^49^.

In this study, we used iPSC-derived MNs harbouring mutations in two different genes associated with ALS, *TARDBP* and *C9ORF72*. *TARDBP* encodes the RNA-binding protein TDP-43, which can act as a transcription repressor and a splicing regulator, and can also contribute to RNA stability and transport^50^,^51^,^52^. Mutations in *TARDBP* are associated with deficits in RNA processing^53^,^54^. Although the exact function of *C9ORF72* remains unknown, its mutant form also appears to cause aberrant RNA processing^5^,^6^,^11^. Thus, defects in RNA processing provide a mechanistic link between *TARDBP* and *C9ORF72*-mediated ALS and may contribute to loss of voltage-activated currents in patient iPSC-derived MNs. In support of this, recent work has shown that the expression of genes involved in neuronal excitability, including those encoding ion channels, is altered in iPSC-derived MNs with *C9ORF72* mutations^12^. Common effects on RNA processing might also in part explain why MNs derived from *TARDBP* and *C9ORF72* iPSC lines exhibited similar pathophysiology; hyperexcitability, followed by a loss of functional output due to a reduction in the magnitude of voltage-activated currents. Alternative interpretations include the possibility that perturbations in ion channels and MN output represent core disease mechanisms common to many forms of ALS, including both familial and sporadic ALS given the demonstration of *TARDBP* and *C9ORF72* mutations in both of these forms of the disease. Further experiments using a variety of patient iPSC lines will be needed to confirm the relevance of our findings to a wide spectrum of ALS cases.

In summary, our study has provided important new insight into early ALS disease mechanisms by investigating pathophysiological changes in iPSC-derived MNs from ALS patients with two different genetic mutations. We have shown that MN output is severely compromised, due to the loss of voltage-activated currents, before any other overt signs of neurodegeneration. These data suggest that ‘functional loss’ of MNs may render the motor system inactive before neurodegeneration of MNs, highlighting the importance of addressing MN function, perhaps by targeting ion channels, when designing new treatment strategies for ALS. Furthermore, our findings demonstrate the usefulness of sensitive physiological studies of human iPSC-derived MNs for future work aiming to develop much needed therapeutics for this devastating disease.

## Methods

### iPSC lines

For this study, we used eight iPSC lines from six individuals: one male M337V TDP-43 ALS patient (two clones; D1 and D3); one female *C9ORF72* ALS patient (one clone, S6); one male *C9ORF72* ALS patient (one clone, R2); two female healthy controls (two clones, R6 and M2; one clone, D6) and one male healthy control (one clone, D9). All participants provided written signed consent to donate their skin sample to derive iPSCs and their use was approved by the Ethics Committee from the King’s College Hospital, a national Medical Research Ethics Committee (MREC). iPSCs were generated from fibroblasts as previously described^9^,^25^. At least four iPSC differentiations were performed for each line (Control: D6=8, D9=6, M2=7, R6=4; *TARDBP*: D1=6, D3=8; *C9ORF72:* S6=4, R2=4), with control and patient iPSC differentiations always running in parallel. iPSCs were established by virally transducing 10^5^ fibroblasts with the Yamanaka reprogramming factors *OCT4*, *SOX2*, *KLF4* and *c-MYC* using either lentiviral (Vectalys) or Sendai (Life technologies) reprogramming kits. Cells were maintained in murine embryonic fibroblast (MEF) media at 37 °C and 5% CO_2_. MEF media consisted of KO-DMEM (Invitrogen), 2 mM L-glutamine (Invitrogen), 10% FBS (Invitrogen) and 1% penicillin/streptomycin (Invitrogen). After 5 days, the cells were split and re-plated as single cells on to an MEF feeder plate. On day 7, media was changed to Knockout serum replacement (KSR) and fibroblast growth factor 2 (FGF2) consisting of advanced DMEM (Invitrogen), 20% serum replacer (Invitrogen), 1 μl ml^−1^ FGF2 (PeproTech), 1% penicillin/streptomycin (Invitrogen), 1% L-glutamine (Invitrogen) and 0.007 μl ml^−1^ 2-mercaptoethanol (Invitrogen). After ~21 days, colonies with compact human embryonic cell-like morphology were expanded and clonal lines were established. Human iPSC lines were maintained long term on CF-1-irradiated mouse embryonic fibroblasts, with KO-DMEM (Invitrogen) supplemented with 20% knockout serum replacement (Invitrogen), 10 ng ml^−1^ basic FGF2 (PeproTech), 1% L-glutamine (Invitrogen), 0.007 μl ml^−1^ 2-mercaptoethanol (Invitrogen) and 1% penicillin/streptomycin (Invitrogen) at 37 °C and 5% CO_2_. For the neural conversion, the cells were transitioned to feeder-free culture conditions in MTESR1 media (Stem Cell Technologies) and passaged three times before use.

All iPSCs used in the study exhibited silencing of the four transgenes used to induce pluripotency with subsequent activation of endogenous OCT4, SOX2 and KLF4 (Supplementary Fig. 3). Pluripotency was confirmed by expression of pluripotency markers OCT4, SOX2, TRA-1–60 and NANOG and RT–PCR with three germ-layer differentiation confirmed by SOX1, Nestin, Brachyury, Eomes, FOXA2 and GATA-4 expression (Supplementary Fig. 3). All clones used had a normal karyotype, with genotyping confirming mutations in *TARDBP* lines and hexanucleotide 5′- GGGGCC -3′ repeat expansions in *C9ORF72* lines shown by repeat prime PCR (Supplementary Fig. 3). RNA foci containing 5′- GGGGCC -3′ hexanucleotide repeat expansions were also revealed in *C9ORF72* lines using fluorescence *in situ* hybridization (FISH; Supplementary Fig. 3).

### iPSC differentiation to a MN lineage

Differentiation of iPSCs into a neuronal and MN lineage was performed using modifications of previously established and validated protocols^9^,^26^. The iPSCs were neuralized to neuroectoderm using dual SMAD inhibition in CDM (50% Iscove’s modified Dulbecco’s medium (Invitrogen), 50% F12, 5 mg ml^−1^ BSA (Europa), 1% chemically defined Lipid 100 × (Invitrogen), 450 mM monothioglycerol (Sigma), 7 mg ml^−1^ insulin (Roche), 15 mg ml^−1^ transferrin (Roche), 1% penicillin/streptomycin), supplemented with 1 mM N-acetyl cysteine (Sigma), 10 μM Activin Inhibitor (R&D Systems) and 2 μM Dorsomorphin (Merck Millipore)). This medium was changed every 2–3 days for 4–10 days. Neurospheres were patterned to a caudal, spinal cord identity in CDM, 1 mM N-acetyl cysteine, 5 ng ml^−1^ FGF (PeproTech)/heparin (Sigma; 20 μg ml^−1^) and 0.1 μM retinoic acid (Sigma) for 4–10 days, changing medium every 2–3 days. Caudalized neural stem cells were ventralized in the presence of Advanced DMEM Nutrient Mixture F12 (Invitrogen), 1% penicillin/streptomycin, 0.5% GlutaMAX, 1% B-27, 0.5% N-2 supplement, 5 ng ml^−1^ FGF+Heparin, 1 μM retinoic acid and 1 μM purmorphamine (Merck Millipore) for 4–10 days, changing every 2–3 days. The FGF/Heparin was removed from the medium and replaced with 0.5 μM purmorphamine, with the cells cultured for another 4–14 days, changing the medium every 2–3 days. Caudalized and ventralized neural stem cells were transitioned to MN maturation medium containing advanced DMEM/F12, 1% penicillin/streptomycin, 0.5% B-27, 0.5% N-2 supplement, 2 ng ml^−1^ Heparin, 10 ng ml^−1^ brain- derived neurotrophic factor (R&D systems), 10 ng ml^−1^ glial cell line-derived neurotrophic factor (R&D systems), 10 μM forskolin (R&D systems), 0.1 μM retinoic acid and 0.1 μM purmorphamine for 2–6 weeks, changing the medium every 2–3 days. MN progenitors were next dissociated with the Papain Dissociation System (Worthington Biochemical), plated at 50 × 10^3^ cells per well in 24-well plates, with 13 mm glass coverslips coated with poly-ornithine (Sigma), laminin (Sigma), fibronectin (Sigma) and Matrigel (BD Biosciences). Plate down medium consisted of Neurobasal medium (Invitrogen), 1% penicillin/streptomycin, 0.5% GlutaMAX, 0.5% B-27, 0.5% N-2 Supplement, 20 ng ml^−1^ basic FGF, 1 μM retinoic acid, 1 μM purmorphamine, 1 μM mouse Smo agonist SAG (Merck Millipore)^26^. Twenty-four hours post plating, 20 ng ml^−1^ ciliary neurotrophic factor (R&D systems), 10 ng ml^−1^ glial cell line-derived neurotrophic factor and 10 μM forskolin, which promotes cell cycle exit, were added, and this medium was used until day 14, feeding every 3 days. From day 14, RA, SAG, purmorphamine and forskolin was removed from the medium, with cells then maintained for up to 10 weeks. As an indication that neuronal differentiation was successful, with few mitotic cells present, we performed immunostaining for the mitotic marker Ki67 (3–4 weeks post plating). Less than 7% of cells expressed Ki67 in control and patient iPSC-derived cultures.

### Immunofluorescence

Cells were fixed in 4% (wt/vol) paraformaldehyde for 10 min, permeabilized in 0.1% Triton X-100 at room temperature for 10 min and blocked in 3% (vol/vol) goat or donkey serum for 45 min. They were then incubated in primary antibodies for 45 min (Supplementary Table 2) followed by secondary antibodies for 20 min (Alexa Fluor dyes, Invitrogen). The nuclei were counterstained with DAPI (4',6-diamidino-2-phenylindole, Sigma) for 5 min and coverslips were mounted on slides with FluorSave (Merck). Fluorescent imaging was performed on fields of view containing uniform DAPI staining using an Axioscope (Zeiss) microscope. Images were processed with Axiovision V 4.8.1 (Zeiss) and immunolabelled cells counted manually by a blinded observer within ImageJ64 (v 1.47) software. Individual cells were first chosen based on DAPI staining of nuclei before immunolabelling was assessed. Positive staining was defined as a signal clearly above the background fluorescence present in areas devoid of cells.

### Repeat primed–PCR

Repeat primed–PCR was used to confirm the presence or absence of the *C9ORF72* hexanucleotide repeat expansion in control or carrier neurons. PCR amplification was carried out using Multiplex Mastermix (Qiagen); 7% dimethylsulfoxide, 0.6 M Betaine, 7.6 μM Primer F (5′- CTGTAGCAAGCTCTGGAACTCAGGAGTCG -3′), 3.6 μm Primer Repeat R (5′- TACGCATCCCAGTTTGAGACGCCCCGGCCCCGGCCCCGGCCCC -3′), 11.6 μM Tail R (5′- TACGCATCCCAGTTTGAGACG -3′), 8 μM 7-deaza-2′-dGTP and 200 ng DNA. Cycling conditions were performed as per the manufacturer’s recommendations, except for the annealing temperature, which was 68 °C for 15 cycles, then 60 °C for a further 20 cycles. PCR products were separated on an ABI 3130 × l analyser (Life Technologies) and data were analysed using GeneMarker software (Soft Genetics).

### RNA fluorescence *in situ* hybridization

FISH analysis was performed using an Alexa 546-conjugated (GGCCCC)_4_ oligoneucleotide probe (IDT). Briefly, cells on glass coverslips were fixed in 4% paraformaldehyde for 30 min, permeabilized in 70% ethanol at 4 °C, incubated with 50% formamide/2 × SSC for 10 min at room temperature and hybridized for 2 h at 37 °C with the oligoneucleotide probe (0.16 ng μl^−1^) in hybridization buffer consisting of 50% formamide, 2 × SSC, 10% dextran sulphate, yeast transfer RNA (1 mg ml^−1^), salmon sperm DNA (1 mg ml^−1^) and 0.2% Tween-20. The cells were washed twice with 50% formamide/1 × SSC for 30 min at 37 °C and once with 2 × SSC at room temperature for 30 min. Immunostaining was performed as described above.

### Cell viability assays

Cell counts were performed, with the observer blinded to cell type, on 20 IR-DIC images for each cell type (control, *TARDBP* and *C9ORF72*) at each 2 week time point throughout the 10 weeks cells were maintained. Infrared-DIC images were chosen at random from a database of images (× 40 magnification) that were obtained whenever whole-cell patch-clamp recordings were attempted. Cell counts were fitted with a negative binomial generalized linear model and the effect of time assessed with a likelihood ratio test.

For LDH assays, cell culture medium was collected from control and patient-derived lines, which were differentiated in parallel to enable direct comparisons. Medium was collected twice per week across weeks 3–10 post plating from *TARDBP* (D1 and D3), *C9ORF72* (S6: 2 experiments and R2) and control lines (D6: 2 experiments, M2 and R6). LDH activity (mU ml^−1^) was calculated for each cell type using LDH assay kits (Abcam). LDH activity was plotted versus postplating date and compared between cell types using linear models.

Nuclear morphology was assessed, with the observer blinded to cell type, by counting the number of pyknotic nuclei in 40 images taken from control and patient-derived lines at 9–10 weeks post plating. Statistical analysis was performed using a single factor ANOVA followed by Tukey’s *post-hoc* test.

### Electrophysiology

Whole-cell patch-clamp recordings were used to assess the functionality of iPSC-derived MNs. Voltage-clamp mode was used to investigate intrinsic membrane properties. Current-clamp mode was used to investigate the firing properties of MNs. Experiments were carried out in a recording chamber, which was perfused continuously with oxygenated artificial cerebral spinal fluid (aCSF) at room temperature (22–24 °C). Whole-cell patch-clamp recordings were made from cells visualized by IR-DIC microscopy using an Olympus upright BX51WI microscope with a × 40 submersion lens. Patch electrodes (4.0–5.0 MΩ resistance) were pulled on a Sutter P-97 horizontal puller (Sutter Instrument Company, Novato, CA) from borosilicate glass capillaries (World Precision Instruments, Sarasota, FL). Recorded signals were amplified and filtered (4 kHz low-pass Bessel filter) using a MultiClamp 700B amplifier (Axon Instruments, Union City, CA) and acquired at ≥10 kHz using a Digidata 1440A analogue-to-digital board and pClamp10 software (Axon Instruments). Whole-cell capacitance (*C*_m_), input resistance (*R*_N_), series resistance (*R*_S_) and RMP values were calculated using pClamp10 software. Only cells with an *R*_s_<20 MΩ, a RMP more hyperpolarized than −20 mV and *R*_N_>100 MΩ were included in data analysis. *R*_s_ values were not significantly different between control, *C9ORF72* and *TARDBP* lines. Cells were defined as neurons if they had clear fast-inactivating inward currents (≥50 pA). In a subset of experiments, such currents were found to be tetrodotoxin sensitive (data not shown). Recordings from glial cells, which were clearly distinguishable based on their hyperpolarized RMP and absence of inward currents, were excluded from all analyses. An on-line P4 leak subtraction protocol was used for all recordings of voltage-activated currents. Descriptions of voltage- and current-clamp protocols are provided in the Results section.

The aCSF used for all electrophysiological recordings contained the following in mM; 127 NaCl, 3 KCl, 2 CaCl_2,_ 1 MgSO_4_, 26 NaHCO_3_, 1.25 NaH_2_PO_4_, 10 D-glucose (equilibrated with 95% O_2_ and 5% CO_2_ at room temperature, pH 7.45; osmolarity,~310 mOsm). The pipette solution contained (in mM): 140 potassium methane-sulfonate, 10 NaCl, 1 CaCl_2_, 10 HEPES, 0.2 EGTA, 3 ATP-Mg, 0.4 GTP, (pH 7.2–7.3, adjusted with KOH; osmolarity adjusted to ~300 mOsm with sucrose). All drugs were made up as concentrated stock solutions in single-use vials and stored at −20 °C. The final concentrations were achieved by diluting stock solutions in aCSF. Stock solutions of GABA and glycine were made up in distilled water. Stock solutions of glutamate were made up in 0.1 M NaOH. Drug application was via addition to the perfusate.

Electrophysiological data were analysed using Clampfit10 software (Axon Instruments) or Dataview (courtesy of Dr W.J. Heitler, University of St. Andrews). Data from each type of iPSC line (control, *TARDBP* and *C9ORF72*) were pooled for all analyses. Peak Na^+^ currents and peak K^+^ currents (log_10_ transformed), *C*_m_, *R*_N_ and RMP were compared across the three different types of cell lines using factorial ANOVAs with postplating date (in 2 week bins; 3–4, 5–6, 7–8, 9–10) and cell line type (control, *TARDBP* or *C9ORF72*) as factors. *f–I* relationships were compared across cell lines using linear models fitted to the initial, most linear portion of the *f*–*I* relationship (≤50 pA above rheobase). Pair-wise comparisons were made using Tukey’s honest significant difference test, where necessary.

For the purposes of statistical comparisons, spontaneous synaptic activity and action potential generation were classified as either present or absent. These binary data were fitted with a logistic regression using postplating date, transformed with a second-order polynomial and cell line type as factors. Contrasts were made using Wald’s tests and *P*-values adjusted using a Bonferroni correction.

Firing properties were further analysed by assigning cells into four categories (No Spike/Single/Adaptive/Repetitive). These data were then fitted with a multinomial logistic regression using type of iPSC line and either peak K^+^ or peak Na^+^ currents (log_10_ transformed) as factors. Using these models, predicted probabilities for each firing mode were calculated over a range of K^+^ and Na^+^ current magnitudes for all types of iPSC lines. Factors within the multinomial logistic regression were assessed with likelihood ratio tests.

Statistical analyses were performed using SPSS, R and the software packages MASS, multcomp and nnet. All data are presented as mean±s.e.m. Sample sizes were similar to those used in previous studies.

## Additional information

**How to cite this article:** Devlin, A.-C. *et al*. Human iPSC-derived motoneurons harbouring *TARDBP* or *C9ORF72* ALS mutations are dysfunctional despite maintaining viability. *Nat. Commun.* 6:5999 doi: 10.1038/ncomms6999 (2015).

## Supplementary Material

Supplementary Information Supplementary Figures 1-3, Supplementary Tables 1-2, and Supplementary References

## Figures and Tables

**Table 1:** Passive membrane properties

	**Control**	*** TARDBP ***	*** C9ORF72 ***
*C*_m_ (pF)			
Weeks 3–4	10.4±0.4 (196)	12.0±0.5 (103)	8.7±0.3 (102)^##^
Weeks 5–6	11.6±0.3 (322)	11.2±0.4 (171)	10.1±0.5 (69)
Weeks 7–8	10.7±0.3 (216)	16.1±1.1 (96)***	11.9±0.9 (52)^##^
Weeks 9–10	14.0±0.6 (111)	19.7±1.2 (82)***	14.5±0.7 (42)^##^
			
*R*_N_(MΩ)			
Weeks 3–4	727±22	660±33	723±33
Weeks 5–6	774±21	788±27	768±42
Weeks 7–8	774±23	762±38	772±45
Weeks 9–10	709±30	688±39	643±58
			
RMP(mV)			
Weeks 3–4	−45.6±0.8	−39.9±1.0**	−44.8±1.2
Weeks 5–6	−42.8±0.7	−40.4±1.0	−44.2±1.5
Weeks 7–8	−45.6±0.8	−43.2±1.4	−37.9±1.5***
Weeks 9–10	−49.8±1.1	−40.1±1.9***	−45.2±1.9

Values are mean ±s.e.m.; number of cells noted in parentheses.

Significantly different to controls (**P<0.001; ***P<0.0001).

Significant difference between patient lines (##*P*<0.001).
