# Supplementary material for: Human iPSC-derived motoneurons harbouring TARDBP or C9ORF72 ALS mutations are dysfunctional despite maintaining viability
Source: Nat Commun. 2015 Jan 12;6:5999. doi: 10.1038/ncomms6999 (PMC4338554; doi:10.1038/ncomms6999)
Supplement: Supplementary Information — Supplementary Figures 1-3, Supplementary Tables 1-2, and Supplementary References [file ncomms6999-s1.pdf]

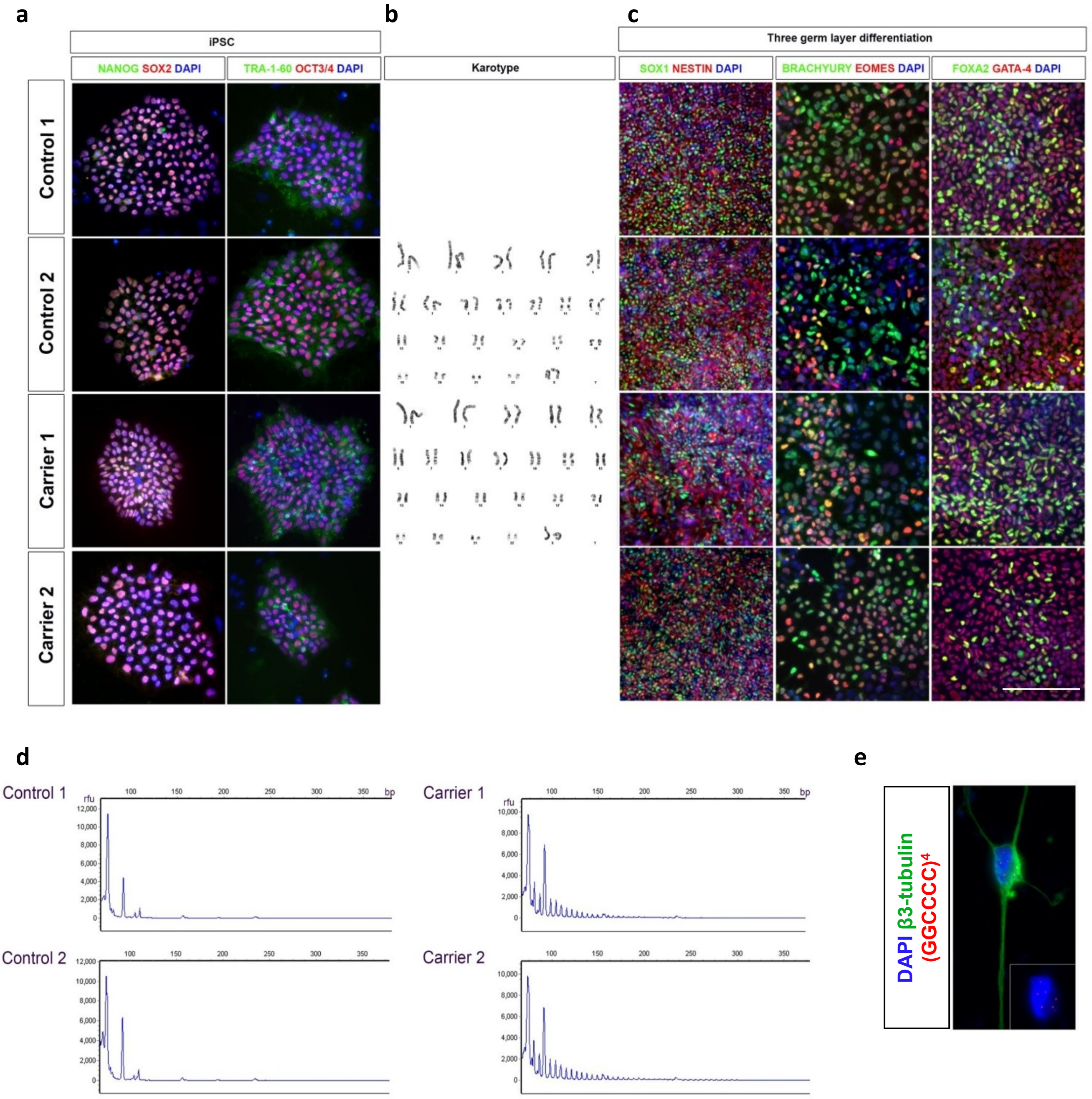

Supplementary Figure 1 : Confirmation of pluripotency and three germ layer differentiation. (a) Immunohistochemical labelling of the pluripotency markers NANOG, SOX2, OCT3/4 and TRA-1-60 in feeder free iPSCs from controls and patients harbouring the *C9ORF72* mutation [for *TARDBP*, see [1]]. (b) Karyotypes of control and patient-derived iPSCs. (c) Immunohistochemical staining of the three germ layers differentiated from control and patient iPSC lines: neuroectoderm (SOX1 and nestin); mesoderm (brachyury and Eomes); and endoderm (FOXA2 and GATA-4; see antibodies Table S1, scale bar =50µm). (d) RT- PCR analysis demonstrating the presence or absence of the *C9ORF72* hexanucleotide repeat expansion. (e) Representative image of GGGGCC RNA foci of the *C9ORF72* hexanucleotide repeat expansion in an iPSC-derived neurons from a patient harbouring the *C9ORF72* mutation. RNA fluorescence in-situ hybridisation (FISH) was carried out using an Alexa546 conjugated $(GGCCCC)_4$ probe.

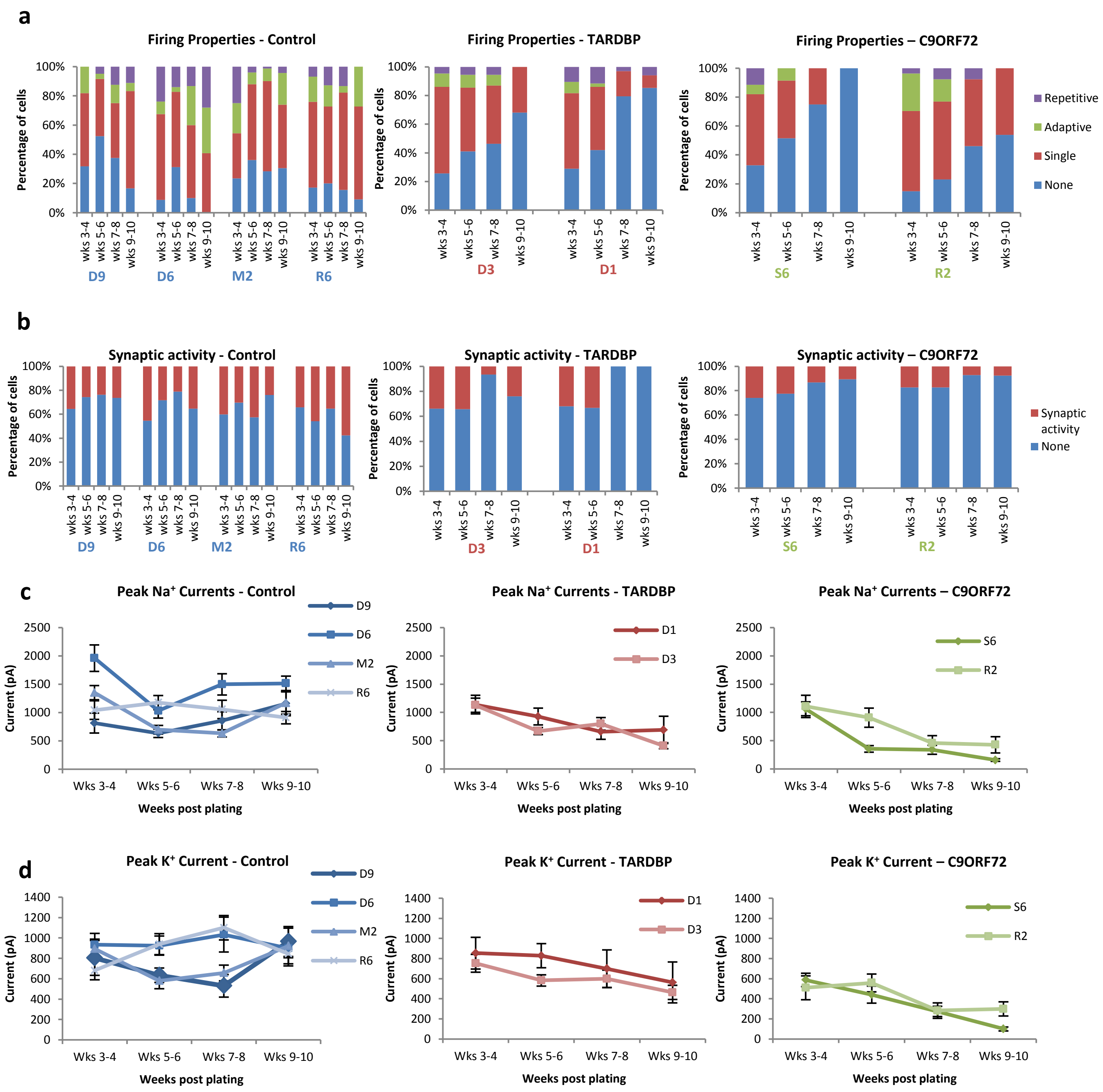

Supplementary Figure 2: Physiological properties of MNs derived from individual iPSC lines
(a) Firing properties for each of the 8 iPSC lines utilised: (controls lines: D9, D6, M2 and R6; TARDBP lines: D1 and D3; C9ORF72 lines: S6 and R2). (b) Percentage of cells in which synaptic activity was present for each iPSC line. (c) Peak Na+ currents and (d) peak K+ currents in each iPSC line from 3- 10 weeks post-plating (see Supplementary Table 2 for sample sizes).

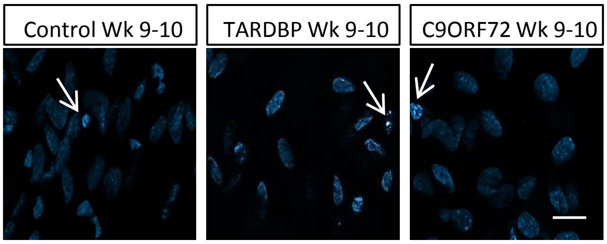

Supplementary Figure 3: Pyknotic nuclei morphology
Examples of pyknotic nuclei (arrows) revealed using DAPI staining at 9-10 weeks post-plating in control, TARDBP and C9ORF72 iPSC-derived MN cultures (scale bar =10µm).

Supplementary Table 1: Primary antibodies used

Antibody	Host	Company	Concentration
**B3-Tubulin**	Mouse monoclonal	Sigma	1:1000
**HB9**	Mouse monoclonal	Developmental Studies Hybridoma Bank	1:250
**GFAP**	Rabbit polyclonal	Dako	1:500
**SMI-32**	Mouse monoclonal	Covance	1:250
**Ki67**	Mouse monoclonal	Dako	1:200
**SOX1**	Goat polyclonal	R&D systems	1:100
**Nestin**	Mouse monoclonal	Milipore	1:100
**Brachyury**	Goat polyclonal	R&D systems	1:100
**Eomes**	Rabbit polyclonal	Abcam	1:600
**Fox A2**	Goat polyclonal	R&D systems	1:100
**GATA-4**	Mouse monoclonal	Santa Cruz	1:100

Supplementary Table 2: Electrophysiology sample sizes

a

D6 Control		C_m (pF)	R_N (MΩ)	RMP (mV)
	Wks 3-4	12.7 ± 0.8	519.7 ± 40.1	-45.7 ± 1.6
	Wks 5-6	13.8 ± 0.7	756.9 ± 49.7	-41.6 ± 1.4
	Wks 7-8	12.9 ± 1.2	672.7 ± 60.0	-47.4 ± 2.3
	Wks 9-10	14.6 ± 0.9	619.3 ± 52.7	-50.4 ± 1.7
D9 Control				
	Wks 3-4	9.4 ± 1.1	784.1 ± 62.1	-40.7 ± 2.2
	Wks 5-6	10.5 ± 0.4	793.5 ± 38.0	-41.2 ± 1.1
	Wks 7-8	12.3 ± 0.7	685.4 ± 63.5	-44.5 ± 2.1
	Wks 9-10	10.7 ± 0.8	784.2 ± 84.1	-48.6 ± 2.1
R6 Control				
	Wks 3-4	9.3 ± 0.4	831.2 ± 27.3	-46.5 ± 1.3
	Wks 5-6	10.9 ± 0.6	721.7 ± 40.5	-48.1 ± 1.4
	Wks 7-8	9.8 ± 0.6	749.5 ± 48.7	-49.1 ± 1.6
	Wks 9-10	12.3 ± 0.9	801.9 ± 47.1	-49.5 ± 1.8
M2 Control				
	Wks 3-4	9.8 ± 0.5	758.9 ± 35.7	-46.9 ± 1.4
	Wks 5-6	10.9 ± 0.5	827.7 ± 38.1	-40.8 ± 1.6
	Wks 7-8	9.7 ± 0.4	859.4 ± 33.2	-45.5 ± 1.3
	Wks 9-10	18.0 ± 1.7	652.7 ± 68.1	-50.45 ± 3.2
D1 TARDBP				
	Wks 3-4	13.6 ± 0.9	652.1 ± 52.3	-39.0 ± 1.7
	Wks 5-6	12.9 ± 0.8	773.3 ± 51.3	-40.4 ± 1.7
	Wks 7-8	24.3 ± 2.4	602.7 ±72.7	-43.6 ± 2.5
	Wks 9-10	28.4 ± 1.6	503.6 ± 51.9	-43.9 ± 3.9
D3 TARDBP				
	Wks 3-4	10.5 ± 0.5	668.8 ± 43.4	-40.9 ± 1.3
	Wks 5-6	10.4 ± 0.4	795.9 ± 32.3	-40.4 ± 1.3
	Wks 7-8	11.5 ± 0.6	850.7 ± 41.3	-43.1± 1.6
	Wks 9-10	12.8 ± 1.1	834.0 ± 48.3	-37.2 ± 1.6
S6 C9ORF72				
	Wks 3-4	9.4 ± 0.4	734.7 ± 41.8	-45.3 ± 1.3
	Wks 5-6	11.5 ± 0.6	761.9 ± 61.0	-43.7 ± 1.7
	Wks 7-8	11.8 ± 0.9	769.5 ± 52.3	-39.0 ± 1.9
	Wks 9-10	15.9 ± 0.9	520.8 ± 69.7	-47.1 ± 2.2
R2 C9ORF72				
	Wks 3-4	7.5 ± 0.4	694.5 ± 54.1	-43.6 ± 2.4
	Wks 5-6	8.1 ± 0.6	777.9 ± 55.7	-44.9 ± 2.7
	Wks 7-8	12.2 ± 2.6	781.51 ± 93.0	-35.1 ± 2.3
	Wks 9-10	11.6 ± 1.1	887.7 ± 75.0	-41.4 ± 3.5

b

D6 Control		V-clamp No. Cells	I-clamp No. Cells
	Wks 3-4	53	46
	Wks 5-6	85	64
	Wks 7-8	33	30
	Wks 9-10	34	32
D9 Control			
	Wks 3-4	33	22
	Wks 5-6	97	82
	Wks 7-8	38	24
	Wks 9-10	19	18
R6 Control			
	Wks 3-4	35	29
	Wks 5-6	74	55
	Wks 7-8	51	45
	Wks 9-10	33	33
M2 Control			
	Wks 3-4	77	68
	Wks 5-6	66	50
	Wks 7-8	94	81
	Wks 9-10	25	23
D1 TARDBP			
	Wks 3-4	50	38
	Wks 5-6	54	43
	Wks 7-8	34	34
	Wks 9-10	35	34
D3 TARDBP			
	Wks 3-4	53	43
	Wks 5-6	118	90
	Wks 7-8	61	54
	Wks 9-10	47	44
S6 C9ORF72			
	Wks 3-4	73	61
	Wks 5-6	40	35
	Wks 7-8	38	36
	Wks 9-10	28	28
R2 C9ORF72			
	Wks 3-4	29	27
	Wks 5-6	29	26
	Wks 7-8	14	13
	Wks 9-10	13	13

c

	Synaptic events No. cells	*f*-I relationship No. cells
D6 Control	11	10
D9 Control	13	3
R6 Control	5	24
M2 Control	9	25
D1 TARDBP	11	7
D3 TARDBP	16	12
S6 C9ORF72	8	15
R2 C9ORF72	1	4

Supplementary References:

[1] Bilican, B. *et al.* Mutant induced pluripotent stem cell lines recapitulate aspects of TDP-43 proteinopathies and reveal cell-specific vulnerability. *Proc. Natl. Acad. Sci. U. S. A.* **109,** 5803–8 (2012).
